# Prevalence and Associated Factors of Depression among Orphan Adolescents in Addis Ababa, Ethiopia

**DOI:** 10.1155/2018/5025143

**Published:** 2018-08-26

**Authors:** Mekdes Beze Demoze, Dessie Abebaw Angaw, Haregwoin Mulat

**Affiliations:** ^1^Department of Psychiatry, College of Medicine and Health Science, University of Gondar, Ethiopia; ^2^Department of Epidemiology, Institute of Public Health, Jimma University, Jimma, Ethiopia

## Abstract

**Background:**

Orphan adolescents are a special group of people who are generally deprived and prone to develop psychiatric disorder even if reared in a well-run institution.

**Objective:**

To assess the prevalence and associated factors of depression among orphan adolescents living in Addis Ababa orphan centers, Addis Ababa, Ethiopia.

**Method:**

A cross-sectional study was conducted in 2016 among 453 orphan adolescents in Ethiopia. All orphan adolescents who were living in the selected orphan centers were included in the study. The data was collected by interviewing the orphan adolescents at the orphan center by using a structured questionnaire. Kocher adolescent depression scale and MSPSS scale were used to measure orphan level of depression and their perceived social support. After appropriate coding, the collected data had been entered into EPI info version 7 and it was exported to SPSS version 20 for further analysis. The OR with 95% CI was used to measure association and p value < 0.05 was used as statistically significant value in multivariable binary logistic regressions.

**Result:**

The overall prevalence of depression among the orphan adolescents was found to be 36.4%. The majority of the respondents, 302 (66.7%), were within the age range of 15-19 years. Perceived social support (OR 5.86; 95% CI 3.47, 9.91), community discrimination (OR 2.68; 95% CI 1.58, 4.56), length of stay (OR 1.90; 95% CI 1.08, 3.35), age of entrance (OR 2.21; 95% CI 1.32, 3.69), and presence of visitors (OR 3.62; 95% CI 2.06, 6.37) were the main variables associated with depression.

**Conclusion:**

The prevalence of depression among orphan adolescents was found to be high. Low level of social support, higher length of stay, community discrimination, the presence of visitors, and younger age of entrance were statistically significant variables to develop depression.

## 1. Introduction

Depression is a disorder that is defined by certain emotional, behavioral, and thought patterns. Adolescent depression is leveled as depressed mood, depressive syndrome, and clinical depression. Depressed mood is sadness at various times in response to unhappy situation. Depressive syndrome is experiencing anxiety with other symptoms such as feeling sad, lonely, unloved, and worthless. Clinical depression is manifestation of five or more depressive symptoms lasting continuously for two weeks and impairing current functioning. Depression is underrecognized among adolescents because depressive symptoms are considered a familiar part of adolescents' experience [1].

Orphan adolescents are a special group of people who are generally deprived and prone to develop psychiatric disorder even if reared in a well-run institution [2]. And they are the most serious socioeconomic and developmental challenge victims in developing countries. They are frequently accompanied with multidimensional problems [3]. Adolescents in particular are at increased risk for unresolved or complicated bereavement because of their developmental vulnerability and emotional dependency and have psychological problems that can affect their present and future life [4].

The death of one or both parent has a profound and lifelong impact on the psychological well-being of children. Common reaction in children to the death of a parent includes depression, hopelessness, anxiety, and fear of being alone that can further jeopardize children's prospect [5–7]

Orphan had a greater risk of anxiety, depression, and anger than nonorphan. Furthermore, an orphan had significantly higher scores than nonorphans [7, 8]. On the other hand, children adolescent orphaned by AIDS were more likely to report symptoms of depression, peer relationship problems, and posttraumatic stress than nonorphaned children [9–12].

Different studies in different countries revealed that the range of 2.6% to 19.4% of orphan adolescents had depressive symptom [13–24].

In Sub-Saharan Africa depression in childhood and adolescent ranges between 7.6% and 34.7%. For example, depression in Uganda was 7.6 %, in Egypt it was 20%, and in Ethiopia it was 25.3- 34.7% [25–28].

Different studies revealed that having older age, being female, and increased educational status were important variables which have a significant effect on developing depression [29–35].

Different works of literature found that orphanage with low social support, discrimination, and having no biological relatives suffered more from depressive disorder [36–39]. A study done in Ethiopia (Mekelle) in 2013 showed that community discrimination and friend discrimination were highly significant factors with depressive disorder [40].

Orphan adolescents are special people who need care, support, and protection from the society and they are the most serious socioeconomic victims. Therefore, the finding of this study will have a good contribution to the area of social and psychological knowledge building and it will provide fresh information on depression and associated factors. On the top of this, it will also help those involved in this area to identify children who are at low level of psychological well-being and to prevent the situation.

The aim of the current study was to assess prevalence and associated factors of depression among orphan adolescents living in Addis Ababa orphan centers.

## 2. Methods

### 2.1. Study Design, Setting, Participants, and Sampling Procedure

A cross-sectional study was conducted in Addis Ababa orphan centers from May 01 to 30, 2016. There were 30 organizations that have given care and support to orphans in Addis Ababa. Twenty percent of them named as Human Capital Group Homes, Saddharta, Gift of Love 1, Missionary of Charity, Lesperance, and Gift of Love 2 were randomly selected for the study.

All orphan adolescents aged 10-19 who were living in the selected orphan centers participated in the study. The sample size was calculated using a single population proportion formula for a descriptive part and EPI info version 7 for associated factors. A complete survey was conducted in the 6 selected orphan centers to get lists of orphans ahead of data collection. In the survey, a total of 465 orphans were identified. Since the calculated sample size (449) was nearly similar to the number of orphans (465) in the selected orphan centers, all of the adolescent orphans were included in the study.

### 2.2. Data Collection Tools, Quality Control Issues, and Study Variables

A structured interviewer administered questionnaire containing Kocher adolescent depression scale to measure feeling of depression. It contains 6 items; each item score ranged from zero to three. Scores of 6 and above on the subscale were classified as having depression [36].

The data was collected by interviewing all orphan adolescents who fulfilled the criteria at their orphan centers. Six psychiatric nurses and one supervisor were assigned to data collection. Before the data collection, two-day training had been given for the data collectors and for the supervisors. The process of data collection was checked by supervisors through random spot checking of the questionnaire to ensure the reliability of the data.

The questions were first prepared in English language and then translated to Amharic language and finally back translated to English to keep its consistency. Pretest was conducted on 44 respondents (5% of total sample size) before five days of data collection in Addis Ababa nonselected orphan centers, and necessary correction was taken after the pretest done on the questionnaires

### 2.3. Data Processing and Analysis

After appropriate coding, the collected data had been entered into EPI info version 7 and it was exported to SPSS version 20 for analysis. Descriptive statistics like frequency, percentage, and cross-tabulation had been computed. Dependent variables and independent variables were entered into bivariate logistic regression in order to detect their significant association. All variables with p value less than 0.25 were entered into multivariable logistic regression in order to identify interaction between variables and to control potential confounders. A p value less than 0.05 had been declared as significant statistical relationship dependent and independent variable. Variables such as sociodemographic, individual, and social factors were included in the study.

## 3. Result

### 3.1. Sociodemographic Characteristics of Respondents

Among 465 eligible orphan adolescents 453 orphan adolescents participated with response rate of 97.4%. The majority of the respondents, 114 (38%), were within the age range of 15-19. From the total respondents 246 (54%), 199 (43%), and 8 (1.8%) were from primary school, high school, and college, respectively (Table 1). Regarding age of entrance into the orphanage 272 (60%) of respondents join the orphanage at the age between 0 and 5 and 181 (40%) at the age 6 and above (Table 2).

### 3.2. Prevalence of Depression among Orphan Adolescent

**Figure 1:**
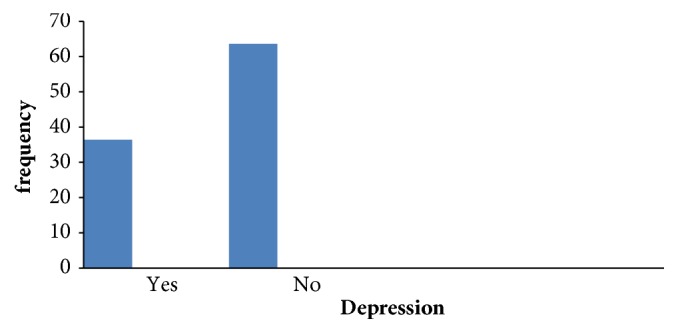
Prevalence of depression among orphan adolescent in Addis Ababa, Ethiopia, Jun 2016.

The overall prevalence of depression among the respondents was found to be 36.4% (CI: 32.1, 40.8) (Figure 1).

### 3.3. Factor Associated with Depression

After controlling all variables the final model consists of 5 variables which contributed to depression outcome significantly with p value <0.05. Orphan adolescents who were entered into the orphanage before 5 years were (OR=1.9; 95% CI: 1.186, 2.994) almost 2 times more likely to be depressed as compared to those who entered after 5 years. Those who have lower level of social support were (OR=5.3; 95% CI=3.2, 8.8) 5.3 times more likely to develop depression as compared to those who have high level of social support. Regarding length of stay those who have higher duration have (OR=1.8; 95% CI=1.019, 3.155) 2 times more chance to have depression. Regarding visitors those who have no visitors in the orphanage have (OR =3.487; 95% CI= 2.081, 5.843) 3.5 times more chance to have depression (Table 3).

## 4. Discussion

In this study the magnitude of depression was 165 (36.4%). This finding was in line with the study done in Addis Ababa (34.7%) [27]. On the other hand, it was higher than the prevalence of depression in USA, 5.7% [13], Sweden, 5.8 % [16], and Bangladesh, 15% [21]. The possible reason for this difference could be using of instrument and difference in society and culture. In the current study the prevalence of depression is also higher than the study done in South Africa, 17% [24], Egypt, 20% [26], and Mekelle, 25.3% [28]. The possible reason for this difference could be the fact that the current study is a result of having high level of community discrimination, low level of social support, and younger age of entrance in the orphanage making them highly vulnerable than the previous study.

The present study found that depressive disorder has statistically significant association with social support. The result presented the fact that orphans having low level of social support were almost six times more likely to develop depression than orphan who has moderate level of social support. This finding was supported by a study done in Mekelle [40] which states that low level of social support was related to a variety of psychological, social, and academic related outcomes. On the other hand, depressive disorder also had statistically significant association with community discrimination. An orphan who has community discrimination was 2.68 times more likely to develop depression as compared to those who do not have community discrimination, and this finding was also supported by similar studies in India [37]. This might be due to the fact that the perception of adolescent about the society will make them lonely.

Depression is also predicted by length of stay in the orphanage and the result shows that there is significant relationship between depression and higher length of stay in the orphanage and that orphan has 1.9 times more chance to have depressive disorder and this result is supported by other studies done in Dhaka [36]. This might be due to the reason that when a child stays longer in the restricted area, they cannot get their family whenever they want and they will be hopeless when they stay longer that they may think themselves lonely. On the other hand, age of entrance in the orphanage had significant association with depressive disorder and orphans who join the orphanage before 5 years of age were 2.2 times more likely to develop depression than those who join the orphanage after 6 years of age. Similar study done in Dhaka also states that early age of entrance in the orphanage was significantly associated with depression [36]. This might be due to the reason that the absence of early parental rearing may contribute as a risk factor to high prevalence of depressive disorder.

The other factor which has significant association with depression is the prescience of visitors in the orphanage centers. Adolescent orphan who has no visitors in the orphanage was 3.6 times more likely to have depression than that who has visitors in the orphanage. The possible reason might be feeling of loneliness or feeling of having no one in the orphanage makes the orphans vulnerable to be depressed. The other factors which are age, sex, educational status, orphan type, and biological relatives did not show statistically significant relationship with depressive disorder. However, the practical part of the result shows that when age increases depressive disorder also increases, and females were more depressed than males; regarding educational status college students were more depressed than primary and secondary education students.

Regarding orphan type, double type of orphan was more depressed than maternal and paternal type of orphan and finally regarding presence of biological relatives orphan who has no biological relatives in the orphanage is more depressed than orphan who has relatives in the orphanage.

## 5. Limitations of the Study

The probable limitation of this study may be that the instrument was not validated previously in Ethiopia. On the top of this, The participants were taken only from Addis Ababa orphan centers, which limits generalization only to Addis Ababa adolescent orphans. Therefore, a more representative study in the matter should be performed in future.

In conclusion the overall prevalence rate of depression among orphan adolescents was found to be high. Large numbers of orphan adolescents were having emotional and psychological problems which certainly affect their present and future life. Variables such as low social support, community discrimination, higher length of stay, having no visitors, and younger age of entrance were significant factors associated with depression.

## Figures and Tables

**Table 1:** Sociodemographic characteristics of orphan adolescents in Addis Ababa, Ethiopia, Jul 2016 (N-453).

Variables	Category	frequency	%
Age	10-14	151	33.3%
	15-19	302	66.7%
Sex	Male	248	54.7%
	Female	205	45.3%
Religion	Orthodox	234	51.7%
	Muslim	19	4.2%
	Catholic	160	35.3%
	Protestant	37	8.8%
Educational status	Primary	246	54.3%
	High school	199	43.9%
	College	8	1.8%

**Table 2:** Distribution of depression prevalence among orphan adolescents in Addis Ababa orphan centers, Addis Ababa, Ethiopia, 2016 (N=453).

variables	category	Level of depression	
		Non depressed	depressed
age	10-14	100(66.2%)	51(33.8%)
	15-19	188(62.2%)	114(37.7%)
sex	Male	160(64.5%)	88(35.5%)
	female	128(62.4%)	77(37.6%)
Educational status	primary	161(65.4%)	85(34.6%)
	high school	123(61.8%)	76(38.2%)
	college	4(50%)	4(50%)
Age of entrance	0-5	158(58.1%)	114(41.9%)
	6 and above	130(71.8%)	51(28.2%)
Orphan type	maternal	19(63.3%)	11(36.7%)
	paternal	48(64.0%)	27(36.0%)
	double	221(36.5%)	127(36.5%)
Length of stay	0-5	75(73.6%)	27(26.4%)
	6 and above	213(60.7%)	138(39.3%)
Presence of biological relatives	yes	53(64.6%)	29(35.4%)
	no	235(63.3%)	136(36.7%)
Presence of visitors	yes	104(58.8%)	73(41.2%)
	no	184(66.7%)	92(33.3%)
Community discrimination	yes	37(38.1%)	60(61.9%)
	no	251(70.5%)	105(29.5%)
Perceived social support	Low support	77(43.3%)	101(56.7%)
	Moderate support	211((76.7%)	64(23.3%)

**Table 3:** A multivariable logistic regression of associated factors with depression among orphan adolescents in Addis Ababa, Ethiopia, Jul 2016 (N=453).

**variables**	**Category**	**depression**		**Crude OR**	**Adjusted OR**
		**yes**	**no**	**(95**%**CI)**	**(95**%**CI)**
**Age **	10-14	100	51	1	1
	15-19	188	114	1.18(.78,1.79)	.98(.53,1.79)
**sex**	Male	160	88	1	1
	Female	128	77	1.094	.83(.53,1.31)
**Educational status**	Primary	161	85	1	1
	High school	123	76	1.15(.78,1.70)	.22(.04,1.19)
	College	4	4	1.88(.45,7.71)	.24(.04,1.28)
**Age of entrance**	0-5	158	114	1.83(1.12,2.75)	2.21(1.32,3.69)
	6 and above	130	51	1	1
**Orphan type**	Maternal	19	11	1	1
	Paternal	48	27	.972(.40,2.34)	2.03(.809,5.13)
	Double	221	127	.993(.458,2.15)	1.38(.730,2.64)
**Length of stay**	0-5	75	27	1	1
	6 and above	213	138	1.80(1.10,2.93)	1.90(1.08,3.35)
**Presence of biological relatives**	Yes	53	29	1	1
	No	235	136	1.05(.64,1.74)	.82(.43,1.53)
**Presence of visitors**	Yes	104	73	1	1
	No	184	92	.712(.48,1.05)	3.62(2.06,6.37)
**Community discrimination**	Yes	37	60	3.87(2.42,6.19)	2.68(1.58,4.56)
	No	251	105		1
**Perceived social support**	Low	77	101	4.32(2.87,6.50)	5.86(3.47,9.91)
	Moderate	211	64	.1	1

## Data Availability

The data used to support the findings of this study are available from the corresponding author upon request.
